# Proteasome inhibition triggers the formation of TRAIL receptor 2 platforms for caspase-8 activation that accumulate in the cytosol

**DOI:** 10.1038/s41418-021-00843-7

**Published:** 2021-08-05

**Authors:** Christian T. Hellwig, M. Eugenia Delgado, Josip Skoko, Lydia Dyck, Carol Hanna, Alexa Wentges, Claudia Langlais, Cathrin Hagenlocher, Alexandra Mack, David Dinsdale, Kelvin Cain, Marion MacFarlane, Markus Rehm

**Affiliations:** 1 Department of Physiology and Medical Physics, Royal College of Surgeons in Ireland, Dublin 2, Ireland; 2 Centre for Systems Medicine, Royal College of Surgeons in Ireland, Dublin 2, Ireland; 3 Institute of Cell Biology and Immunology, University of Stuttgart, Stuttgart, Germany; 4 Stuttgart Research Center Systems Biology, University of Stuttgart, Stuttgart, Germany; 5 Biochemical Pharmacology, Department of Biology, University of Konstanz, Konstanz, Germany; 6 MRC Toxicology Unit, Cambridge, UK; 7 Stuttgart Center for Simulation Science (SC SimTech), University of Stuttgart, Stuttgart, Germany

**Keywords:** Cell biology, Molecular biology, Experimental models of disease

## Abstract

Cancer cells that are resistant to Bax/Bak-dependent intrinsic apoptosis can be eliminated by proteasome inhibition. Here, we show that proteasome inhibition induces the formation of high molecular weight platforms in the cytosol that serve to activate caspase-8. The activation complexes contain Fas-associated death domain (FADD) and receptor-interacting serine/threonine-protein kinase 1 (RIPK1). Furthermore, the complexes contain TRAIL-receptor 2 (TRAIL-R2) but not TRAIL-receptor 1 (TRAIL-R1). While RIPK1 inhibition or depletion did not affect proteasome inhibitor-induced cell death, TRAIL-R2 was found essential for efficient caspase-8 activation, since the loss of TRAIL-R2 expression abrogated caspase processing, significantly reduced cell death, and promoted cell re-growth after drug washout. Overall, our study provides novel insight into the mechanisms by which proteasome inhibition eliminates otherwise apoptosis-resistant cells, and highlights the crucial role of a ligand-independent but TRAIL-R2-dependent activation mechanism for caspase-8 in this scenario.

## Introduction

The activation of initiator caspase-8 by induced proximity requires the formation of complexes that recruit and locally accumulate procaspase-8 [1, 2]. During canonical extrinsic apoptosis, these complexes form once death ligands, such as TRAIL or CD95/FasL, bind to their cognate plasma membrane receptors and drive their oligomerization [2]. On the cytosolic receptor sides, the caspase-8 recruiting adapter protein FADD then binds to form the death-inducing signaling complex (DISC) that activates caspase-8 [3]. TNFα-induced apoptosis requires the activation of TNF receptors, which, following receptor internalization, results in caspase-8 activation on complexes that contain FADD and RIPK1 [4]. While RIPK1 can support caspase-8 activation as a scaffold, its activity in the presence of sufficient amounts of RIPK3 and MLKL can also drive necroptosis as an alternative cell death outcome [5]. Importantly, signaling towards apoptosis or necroptosis only proceeds if death receptor activation is sufficiently high and if alternative signaling routes towards pro-survival and proliferation responses are inactive or suppressed [2]. In addition, high amounts of cFLIP proteins, inactive homologs of caspase-8, can interfere with efficient caspase-8 activation. More recently, additional caspase-8 activation platforms have been identified. Cytosolic RIPK1-containing complexes that activate caspase-8 can form subsequent to TLR3 activation or genotoxic stress upon depletion of cIAP1/2 [6, 7]. Likewise, caspase-8 can be activated upon thapsigargin-induced ER stress and in response to glucose deprivation [8–10]. In addition, we previously described that proteasome inhibition can activate caspase-8 independent of death ligands, however, without clarifying the location and the molecular mechanism of caspase-8 activation in this setting [11]. Proteasome inhibition, just like many other intracellular stresses, induces the Bcl-2 family-regulated mitochondrial pathway of apoptosis that culminates in the formation of Bax and Bak pores [12]. However, proteasome inhibition also eliminates cells that cannot trigger Bax/Bak-dependent apoptosis, and caspase-8 stabilization is probably crucial to directly activate caspase-3 and drive apoptosis execution by type I signaling [11]. Since proteasome inhibitors such as bortezomib and carfilzomib are nowadays commonly used as anti-cancer therapeutics [13], we here set out to obtain mechanistic insight into the process of caspase-8 activation, as well as its regulation and relevance for apoptosis susceptibility under conditions of proteasome inhibition.

## Materials and methods

### Materials

HCT-116 were from ATCC. HCT116 (Bax/Bak)^−/−^ cells were a gift from R. Youle (NIH, Bethesda, MD), MEF and MEF (Bax/Bak)^−/−^ cells were a gift from S. Oakes (University of California, San Francisco, CA). Cells were authenticated and regularly mycoplam tested. TRAIL was provided by C.R. Rodrigues dos Reis (University of Groningen, Netherlands). Tunicamycin was purchased from Alexis (San Diego, CA), bortezomib from Millenium Pharmaceuticals (Cambridge, MA), MG-132 from Biomol (Plymouth Meeting, PA, USA), MnTBAP, 3-MA, 5-FU, Suc-LLVY, cisplatin, and necrostatin-1 from Calbiochem, z-VAD-fmk from Bachem (St. Helens, UK), Hoechst 33342 from Immunochemistry (UK), Aggresome Staining Kit, and TRAIL-R1:Fc (ALX-522-004-C050), TRAIL-R2:Fc (ALX-522-005-C050), and TNF-R1:Fc (ALX-522-013-C050) neutralizing antibodies from Enzo Life Sciences (Exeter, UK), Coomassie PLUS (Bradford) protein assay kit and Pierce BCA protein assay kit from Thermo Fisher Scientific, RPMI1640 medium, Q-VD-OPh, rotenone, epoxomicin, propidium iodide, ferrostatin-1, necrosulfonamide, FBS and all other chemicals were purchased from Sigma-Aldrich. Q-VD-OPh was added in concentrations of up to 20–50 µM to fully prevent caspase processing and substrate cleavage, where required [6, 7].

### Cell culture, clonogenic survival analysis, and transfections

Cells were cultured in RPMI1640 medium, supplemented with glutamine and 10% FBS, at 37 °C in a humidified atmosphere of 5% CO_2_. For clonogenic survival analysis, defined numbers of cells were seeded into multi-well plates and colony formation frequencies were determined by automated microscopic imaging (IncuCyte S3 Live-Cell Analysis System (Essen BioScience)). Cells were transfected with short interfering RNA or plasmids using Lipofectamine (Thermo Fisher Scientific) according to the manufacturer’s manual. The following short interfering RNA sequences were used: scrambled siRNA (ACU UAA CCG GCA UAC CGG C [dT] [dT]), siRNA ATG5 (ACU CUG GAU GGG AUU G [dT] [dT]), siRNA caspase-8 (AAU UCG GAA GAG CAG CUC C[dT] [dT]), siRNA RIPK1 (#Hs_RIPK1_5, Qiagen, Germany). For TRAIL-R1 depletion, a cocktail of four siRNAs (ON-TARGET plus siRNA, human *TNFRSF10A*−07/−08/−09/−10, Thermo Scientific Dharmacon) was transfected. siRNA targeting PGAM5 was purchased from Ribox Life Science (IBONI siRNA First Human PGAM5 D-001101-IBONI First; 62012000132). The following plasmids were used: pcDNA3.1-SCAT8 [14], pGFP-LC3 was a gift from N. Mizushima (Tokyo Metropolitan Institute of Medical Science, Tokyo, Japan).

### Flow cytometry

Flow cytometric measurements of propidium iodide uptake and FRET disruption were performed on a BD LSRII SORP HTS cytometer (BD Biosciences, NJ, USA) or a MACSQuant Instrument (Miltenyi Biotec). Data were processed using Cyflogic software and Flowing software (Perttu Terho, CyFlo Ltd, Turku, Finland).

### Proteasome activity assay

Proteasome activity was determined by a fluorescent reporter peptide assay for chymotrypsin-like activity, using Suc-LLVY-AMC (N-Succinyl-Leu-Leu-Val-Tyr-7-amino-4-methyl-coumarin) as a reported peptide. In total, 50,000 cells were seeded per well in a 24-well plate 24 h before treatments started. Cells were then harvested and centrifuged at 200×*g* for 3 min. Pellets were resuspended in 310 µl CHAPS lysis buffer (10 mM HEPES, 42 mM KCl, 5 mM MgCl_2_, 0.1 mM EDTA, 0.1 mM EGTA, 1 mM DTT, 0.5% (w/v) CHAPS) and incubated for 5 min at 37 °C on a shaker. From each cell lysate triplicates were prepared by pipetting 50 µl lysate per well into a black 96-well flat bottom plate. The lysates were incubated with 150 µl reaction solution containing 1× reaction buffer (25 mM HEPES, pH 7.4, 0.5 mM EDTA) and 10 µM Suc-LLVY-AMC. Wells containing 50 µl CHAPS lysis buffer and 150 µl reaction solution served to determine autofluorescence. After 5 min incubation in the dark, plates were placed into a 37 °C pre-warmed Tecan plate reader and fluorescence emission at 465 nm was measured every 10 min upon excitation of 360 nm. Fluorescence signals were normalized to protein amounts, as determined using a Coomassie Plus (Bradford) protein assay kit (Thermo Fisher Scientific).

### Subcellular and high molecular weight fractionation

After treatment, cells from four 175 cm^2^ flasks were washed with PBS and lysed in the presence of Complete protease inhibitors (Roche, Sussex, UK) by shearing with a ball-bearing homogenizer. Subsequently, the lysates were sequentially centrifuged at 4 °C with the relative centrifugation force (RCF) of 900 *g* and 1100 *g* (nuclear fraction) for 15 min, 10,000 *g* and 17,000 *g* (mitochondrial fraction) for 30 min, and finally with 100,000 *g* for 1 h to separate the ER fraction from the cytosolic fraction. Protein complexes from cytosolic fractions were separated by size-exclusion chromatography with a Superose 6 HR 10/30 high-resolution column (GE Healthcare) connected to an ÄKTA Purifier protein purification system (GE Healthcare) as described previously [15].

### Immunoprecipitation

High molecular weight protein fractions from cytosolic extracts were incubated with a caspase-8 antibody (Enzo Life Sciences), then with beads containing covalently bound secondary antibodies. Beads were then washed with several volumes of fresh lysis buffer, containing Complete protease inhibitors (Roche), and caspase-8-associated complexes eluted and solubilized directly in SDS sample buffer for subsequent analysis by western blotting [15].

### Western blotting

Total cell extracts or cytosolic fractions from treated cells were lysed in lysis buffer [62.5 mM Tris-HCl (pH 6.8), 10% (v/v) glycerin, 2% (w/v) SDS, Complete protease inhibitors (Roche) in deionized water] at 95 °C for 30 min, then centrifuged to remove cell debris from the soluble proteins. Equal amounts of protein were loaded onto SDS-polyacrylamide gels, separated by electrophoresis using a Mini-PROTEAN Tetra Cell (BIO-RAD), and then blotted onto nitrocellulose membranes (Schleicher & Schuell, Dassel, Germany) using a Trans-Blot Semi-Dry Transfer Cell (BIO-RAD). Membranes were blocked in 5% (w/v) non-fat dry milk in TBS containing 0.1% Tween20 for 1 h or overnight, incubated with primary antibodies, followed by HRP-conjugated secondary antibodies. The following antibodies were used: Actin, ATG5, LC3, and PGAM5 from Sigma, Bak from Santa Cruz, Bax from Upstate, Bid from R&D Systems, PARP, tubulin, TRAIL-R2, and caspase-3 from Cell Signaling Technology, caspase-8 from Enzo Life Sciences, cFLIP from Alexis Biochemicals, porin from Calbiochem, RIPK1, FADD from BD Biosciences, TRAIL-R1 from ProSci Inc., lamin A, VDAC2, calnexin, and LDH from Invitrogen. Secondary antibodies with conjugated HRP against mouse-, rabbit-, or goat-IgG were from Millipore. Membranes were developed using the Immobilon™ western chemiluminescence HRP substrate (Millipore) and chemiluminescence was detected at a depth of 12-bit in the linear detection range of a Fuji LAS 4000 CCD system (Fujifilm UK Ltd., Bedfordshire, UK). For presentation, images were converted to 8 bit and contrast-adjusted with ImageJ or FIJI software (National Institute of Health, USA, http://rsb.info.nih.gov/ij).

### Electron microscopy

Cells were fixed in 2% glutaraldehyde in 0.1 M sodium cacodylate buffer (pH 7.4) at 4 °C overnight and postfixed with 1% osmium tetroxide/1% potassium ferrocyanide for 1 h at room temperature. After fixation, cells were stained en-bloc with 5% aqueous uranyl acetate overnight at room temperature, dehydrated in a series of alcohols, and embedded in Taab epoxy resin (Taab Laboratories Equipment Ltd., Aldermaston, UK). Ultrathin sections were stained with lead citrate and recorded using a Megaview 3 digital camera and iTEM software (Olympus Soft Imaging Solutions GmbH, Münster, Germany) in a Jeol 100-CXII electron microscope (Jeol UK Ltd., Welwyn Garden City, UK).

### Microscopy

Cells were imaged using a Zeiss LSM 710 META inverted microscope (Carl Zeiss) attached to a confocal laser-scanning unit or by using a widefield epi-fluorescence microscope. For aggresome analysis, cells were fixed and stained with the PROTEOSTAT aggresome staining kit (Enzo Life Sciences, Germany). Caspase-8 immunofluorescence staining was performed using a caspase-8 polyclonal rabbit IgG antibody (Thermo Fisher Scientific, Invitrogen). For confocal imaging, LC3-GFP fluorescence (autophagosome labeling) was detected upon 488 nm excitation (emission 493–501 nm). Aggresome fluorescence labeling was excited at 514 nm (emission 582–621 nm). Caspase-8 immunofluorescence staining with the secondary antibody Alexa Fluor 633 was excited at 633 nm (emission 638–747 nm).

### Genome editing

Oligos coding for the guide RNA targeting Exon 1 of TRAIL-R2 (*TNFRSF10B*) were ordered from biomers.net (5′– CACCgCGCTTCGGGGGCCCGGAAA–3′ (forward), 5′–AAACTTTCCGGGCCCCCGAAGCGC–3′ (reverse); Ulm, Germany), annealed, and ligated into the plasmid pSpCas9(BB)−2A-GFP ((PX458), (#48138), Addgene, Watertown, MA, USA), containing the gRNA scaffold, Cas9, and GFP. One day after transfection, GFP-expressing cells were selected by FACS (BD5 FACSAriaTM III, BD Biosciences, Heidelberg, Germany) and clones from single cells were raised. The TRAIL-R2 knockout was verified by western blotting after two to three weeks of culturing. In parallel, control clones from transfection with the empty vector were selected and raised from single cells.

### Time-lapse imaging

Cells were seeded into 96-well plates and left to attach overnight. Cells were then treated as indicated and the plates were placed into an IncuCyte S3 Live-Cell Analysis System (Essen BioScience). Confluency and the amount of PI-positive cells in 3 fields of view per well in 3 wells per treatment were quantified using the semi-automated IncuCyte S3 software (Essen BioScience).

### Statistical analysis

Statistical analysis was performed using GraphPad Prism 9 (GraphPad Software, San Diego, CA, USA). Figure legends contain information on the respective statistical tests.

## Results

### Caspase-8 is the apical caspase activated upon proteasome inhibition in Bax/Bak deficient cells

**Fig. 1:**
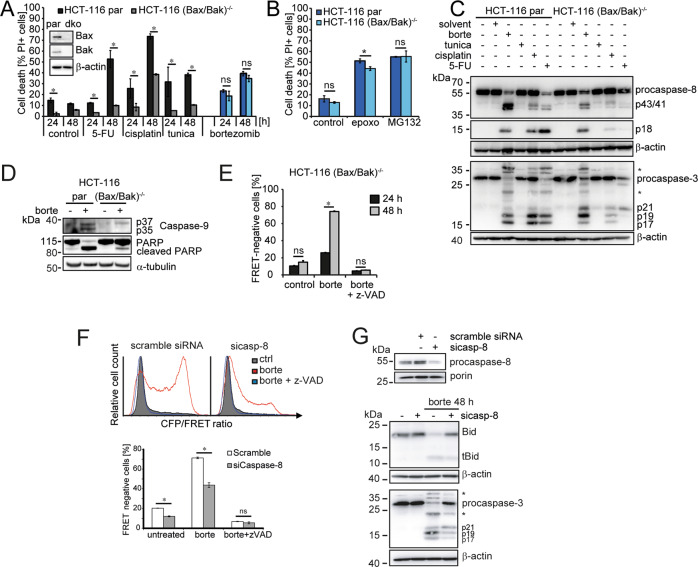
Role of caspase-8 upon proteasome inhibition. **A** Parental HCT-116 (par) and HCT-116 (Bax/Bak)^−/−^ cells were treated with intrinsic apoptosis triggers (100 µg/ml 5-fluorouracil (5-FU), 40 µM cisplatin, 3 µM tunicamycin (tunica), 100 nM bortezomib) as indicated. Cell death was measured by PI uptake. Data are means ± sd from triplicate samples of a representative experiment (*p < 0.05, multiple *t*-test). Immunoblot insert confirms loss of Bax and Bak in HCT-116 (Bax/Bak)^−/−^ cells. β-actin served as loading control. **B** Cell death (PI uptake) upon treatment with alternative proteasome inhibitors (100 nM epoxomicin (epoxo) or 10 µM MG132) for 48 h. Data are means ± sd from triplicate samples (*p* < 0.05, multiple comparisons *t*-test). **C** Cells were treated for 48 h as in (**A**), and cleavage of procaspase-8 and procaspase-3 was analyzed by western blotting. β-actin served as a loading control. Solvent, DMSO control. *Unspecific bands. **D** Cells were treated for 24 h with 100 nM bortezomib as indicated and cleaved caspase-9 and PARP cleavage were analyzed by western blotting. Tubulin served as a loading control. **E** Flow cytometric quantification of IETDase activation in HCT-116 (Bax/Bak)^−/−^ cells treated with 100 nM bortezomib, with or without 20 µM z-VAD-fmk for 24 h or 48 h. IETDase activation was measured using a CFP-IETD-YFP FRET probe. *p < 0.05, ANOVA followed by Sidak’s multiple comparisons test. **F** Quantification of IETD probe cleavage in HCT-116 (Bax/Bak)^−/−^ cells. Cells were transfected with scrambled siRNA or siRNA directed against procaspase-8 (sicasp-8) and treated with 100 nM bortezomib (±50 µM z-VAD-fmk) 24 h after transfection. *p < 0.05 (*t*-tests). **G** Bid and procaspase-3 cleavage were studied in HCT-116 (Bax/Bak)^−/−^ cells transfected as indicated and treated with 100 nM bortezomib. Porin and β-actin served as loading controls. *Unspecific bands.

Studying cells incapable of triggering apoptotic Bax/Bak pore formation (HCT-116 (Bax/Bak)^−/−^), we noted that lack of Bax/Bak substantially reduced or prevented cell death in response to common chemotherapeutics (5-fluorouracil, cisplatin) or the ER stress-inducing agent tunicamycin. In contrast, cell death in response to bortezomib, a therapeutically relevant, highly specific, and rapid inhibitor of proteasomal LLVY-ase activity, was not affected (Figs. 1A and S1A). Similar results were obtained with epoxomicin or MG-132 as alternative proteasome inhibitors (Fig. 1B), and in Bax/Bak-deficient mouse embryonic fibroblasts (Fig. S1B). Despite the absence of Bax/Bak, these cells processed procaspase-8 and -3 (Figs. 1C and S1C). Consistent with Bax/Bak deficiency, only caspase 3-cleaved caspase-9 (p37; resulting from cleavage of procaspase-9 monomers) was detected whereas cleaved caspase-9 generated by dimerization and activation on the apoptosome (p35) was absent (Fig.1D). Caspase-3 activation sufficed to cleave PARP, yet less efficient than in parental cells (Fig.1D). Bortezomib-treated cells cleaved a FRET probe that contains the optimal caspase-8 cleavage motif IETD (Figs. 1E and S1D). Depleting procaspase-8 substantially reduced FRET probe cleavage (Fig. 1F) as well as cleavage of the natural caspase-8 substrates Bid and procaspase-3 (Fig. 1G). Caspase-8, therefore, serves as the apical initiator caspase for apoptotic cell death in response to proteasome inhibition in conditions where the mitochondrial apoptosis pathway is blocked.

### Proteasome inhibition induces apoptosis but not necroptosis, ferroptosis, or ROS-dependent necrosis as the primary programmed cell death modality

**Fig. 2:**
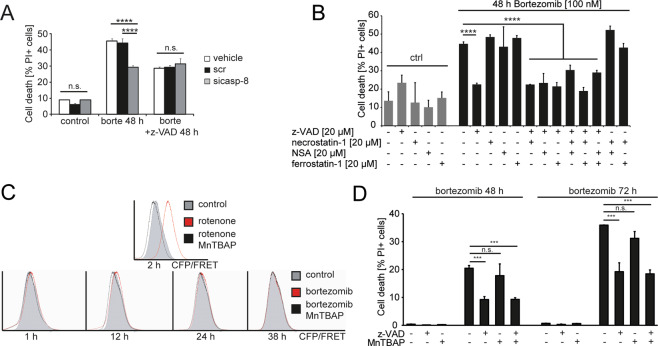
Apoptosis is the only programmed cell death modality induced by proteasome inhibition. **A** HCT-116 (Bax/Bak)^−/−^ cells were mock-transfected (vehicle), transfected with scrambled siRNA (scr) or siRNA directed against caspase-8 (sicasp-8). Twenty-four hours after transfection, cells were treated with 100 nM bortezomib ±50 μM z-VAD-fmk for 48 h. Cell death was measured by PI uptake. Data are means ± sd from triplicate samples (****p < 0.0001, ANOVA followed by Tukey’s multiple comparisons test). **B** Cell death in HCT-116 (Bax/Bak)^−/−^ cells treated with 100 nM bortezomib and co-treated with inhibitors of RIPK1 (necrostatin), MLKL (necrosulfonamide (NSA)), lipid peroxidation, and ferroptosis (ferrostatin). Data are means ± sem from *n* = 4 independent experiments (****p < 0.0001, ANOVA followed by Sidak’s multiple comparisons test). **C** ROS production in response to 50 μM rotenone or proteasome inhibition was measured in HCT-116 (Bax/Bak)^−/−^ cells. 150 μM MnTBAP was added as a scavenger of superoxide and peroxynitrite. ROS were measured by fluorigenic dihydroethidium oxidation. **D** Cell death in HCT-116 (Bax/Bak)^−/−^ cells treated with 100 nM bortezomib and co-treated with z-VAD-fmk or MnTBAP. Cell death was measured by PI uptake. Data are means ± sd from triplicate samples of a representative experiment (***p < 0.001, multiple *t*-test, Bonferroni correction).

We next sought to study which programmed cell death modalities dominate the cellular response to proteasome inhibition. Caspase-8 depletion was as potent as general caspase inhibition by z-VAD-fmk and saved at least one-third of the cells from cell death (Fig. 2A). Any remaining caspase-independent cell death after prolonged proteasome inhibition most likely was necrotic in nature, since contributions by ferroptosis and by RIPK1/RIPK3-dependent or phosphoglycerate mutase family member 5 (PGAM5)/ROS-dependent necroptosis could be excluded. In particular, neither ferrostatin-1 nor necrosulfonamide, inhibitors of RIPK1 and MLKL, respectively, inhibited bortezomib-induced cell death (Fig. 2B). Similarly, depletion of RIPK1 or PGAM5, a phosphatase involved in multiple scenarios of necrotic cell death [16] was ineffective in suppressing cell death (Fig. S1E, F). Ferrostatin, a lipid ROS scavenger, and inhibitor of ferroptotic cell death, as well as the ROS scavenger MnTBAP likewise failed to prevent bortezomib-induced cell death (Fig. 2B–D). These results, therefore, identify caspase-8 as a key initiator caspase for programmed cell death in response to proteasome inhibition.

### Caspase-8 associates with cytosolic protein aggregates following proteasome inhibition

**Fig. 3:**
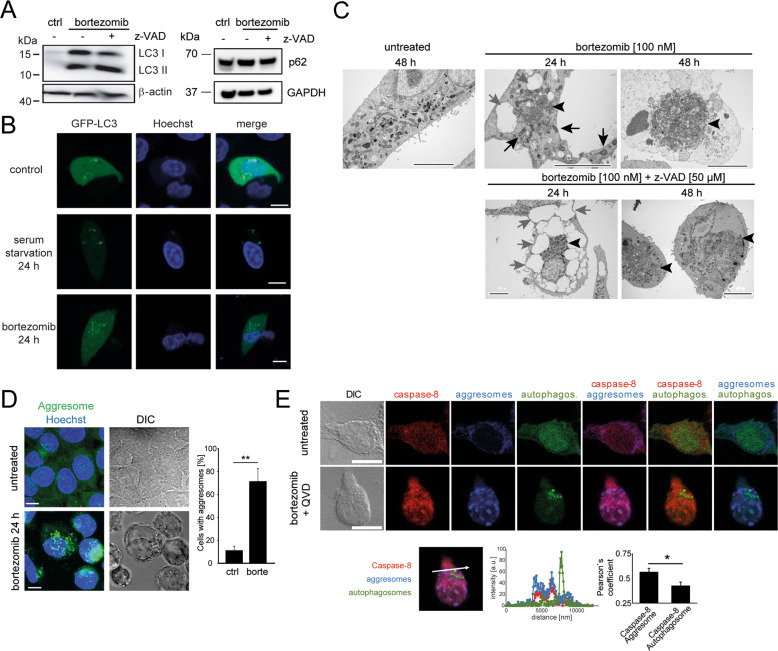
Caspase-8 aggregates and is activated within the cytosol following proteasome inhibition. **A** LC3 I and II as well as p62 amounts in HCT-116 (Bax/Bak)^−/−^ cells treated with 100 nM bortezomib (±50 µM z-VAD-fmk) for 48 h. β-actin and GAPDH served as loading controls. **B** GFP-LC3 distribution in HCT-116 (Bax/Bak)^−/−^ cells treated as indicated. Nuclei were stained with Hoechst 33342. Scale bar = 10 µm. **C** Ultrastructural changes in HCT-116 (Bax/Bak)^−/−^ cells treated as indicated. Condensed mitochondria (black arrows), aggresomes (black arrowheads), dilated ER (gray arrows). Aggresomes appear as optically dense but otherwise largely unstructured subcellular protein accumulations. Scale bars = 5 µm. **D** Aggresome staining in HCT-116 (Bax/Bak)^−/−^ cells treated with 100 nM bortezomib. Nuclei were stained with Hoechst 33342. Scale bar = 10 µm. Data show mean values and sd from n equals three independent fields of view with >30 cells each (**p < 0.01, *t*-test). **E** Localization of caspase-8 and aggresomes in HCT-116 (Bax/Bak)^−/−^ cells expressing LC3-GFP. Cells were treated with 100 nM bortezomib and 5 µM Q-VD-OPh for 48 h, fixed, and immune-stained for aggresomes and caspase-8. Scale bar = 10 µm. Overlay-line scan and Pearson’s correlation analysis demonstrates preferable co-localization of caspase-8 and aggresomes (bar graphs shows mean values and sd from n = 12 cells; *p < 0.05, paired *t*-test).

Caspase-8 activation proceeded independently of the activation of TRAIL-R1, -R2, and TNF-R1 on the cell surface, as demonstrated in experiments using blocking antibodies (Fig. S2A, B). We, therefore, studied if structures other than canonical death ligand-induced signaling platforms could be associated with the caspase-8 accumulation and, by extension, probably activation. Previous work in Bcl-2 overexpressing cells [11] indicated that caspase-8 activation might rely at least in part on autophagy or the presence of autophagy regulators. Also, other studies linked components of the autophagy machinery to non-canonical caspase-8 activation [17–20]. In Bax/Bak deficient cells, microtubule-associated protein 1A/1B-light chain 3 (LC3) accumulated as both LC3-I and –II upon proteasome inhibition, indicating that autophagy may be induced but that autophagic flux (i.e., the conversion of LC3-I to –II) might be impaired (Fig. 3A). The amounts of p62, a common autophagy receptor protein, remained unchanged, likewise suggesting that overall autophagic flux might not necessarily be affected (Fig. 3A). Changes in the subcellular fluorescence distribution of a GFP-LC3 fusion protein supported this conclusion. While serum starvation, a *bona fide* inducer of autophagy, resulted in the loss of cytosolic LC3 fluorescence and formation of punctae representing autophagosomes, large amounts of LC3 remained cytosolic upon proteasome inhibition (Fig. 3B). Inhibiting autophagy with 3-methyadenin or depletion of Atg5 expression only moderately affected caspase-8 processing (Fig. S2C, D). Ultrastructural analyses identified only limited accumulation of autophagosomes or autolysosomes in response to proteasome inhibition but highlighted mitochondria with a dense matrix and swollen cristae, a severely dilated ER, and notably a marked increase in the number of aggresomes (Fig. 3C). Aggresomes likewise were observed by fluorescence-based imaging when staining with a molecular rotor dye that becomes fluorescent when intercalating into the cross-beta spine of quaternary protein structures that are found in aggregated proteins (Fig. 3D). Co-localization studies demonstrated that preferably aggresomes rather than autophagosomes co-localized or associated with caspase-8, as indicated by a significantly higher correlation coefficient (Fig. 3E). Overall, this indicates that caspase-8 preferentially associates with cytosolic protein aggregates following proteasome inhibition.

### Proteasome inhibition induces the formation of TRAIL-R2 containing caspase-8 activation complexes of high molecular weight that accumulate in the cytosol

**Fig. 4:**
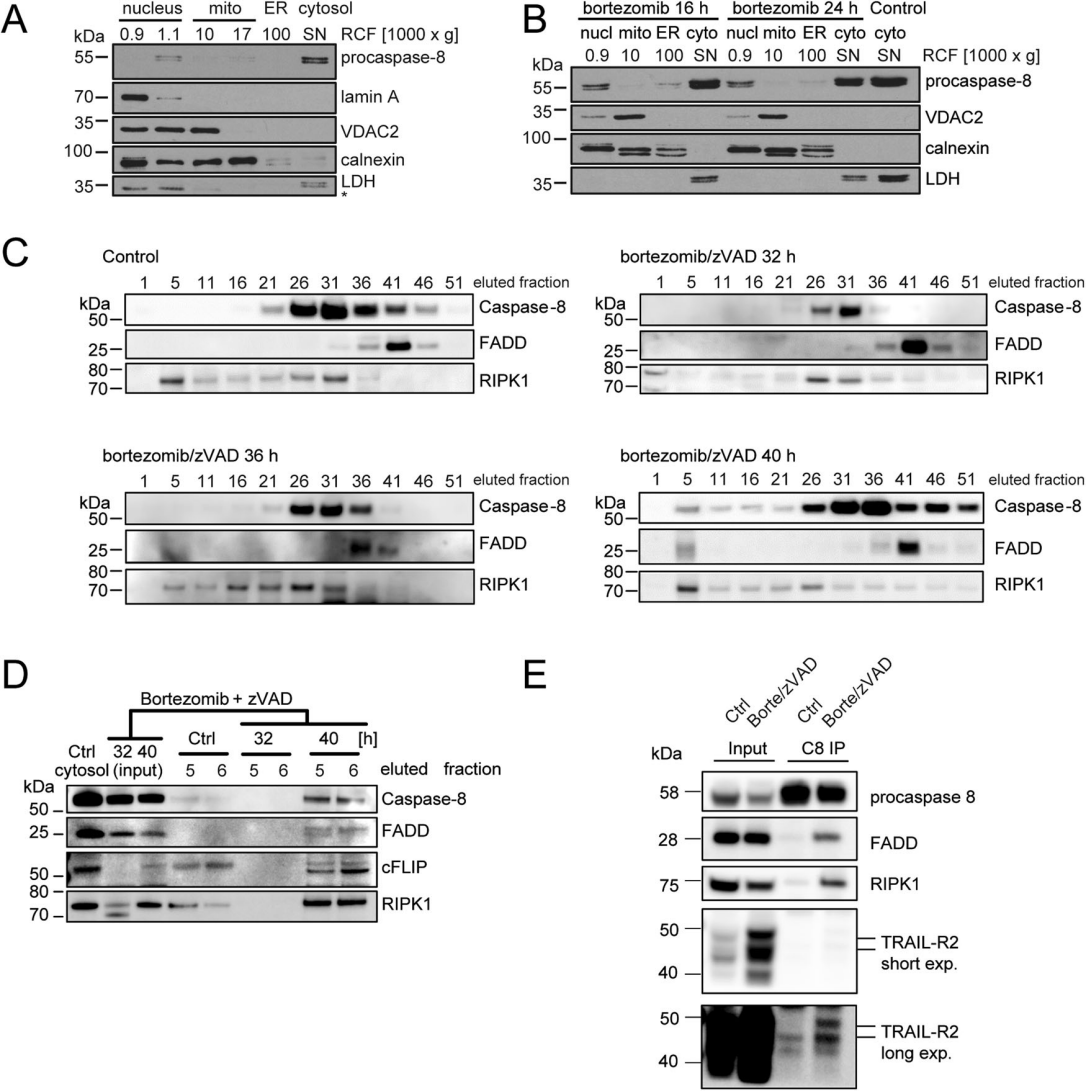
Proteasome inhibition induces the formation of RIPK1-deficient, cytosolic caspase-8 activation complexes with high molecular weight. **A**, **B** Sub-cellular fractions of whole-cell extracts of HCT-116 (Bax/Bak)^−/−^ cells were separated by centrifugation steps (900 to 100,000×*g* RCF) and the localization of caspase-8 was analyzed by western blotting. Lamin A, VDAC2, calnexin, and LDH served to validate fractionation. Cells were untreated (**A**) or treated with 100 nM bortezomib for 16 h or 24 h (**B**). SN, supernatant after 1 h centrifugation at 100,000×*g* RCF (cytosolic fraction). **C**, **D** Soluble proteins in cytosolic fractions were separated according to their molecular weight and the presence of procaspase-8, FADD, cFLIP, and RIPK1 were analyzed by western blotting. Cells were left untreated or treated with 100 nM bortezomib and 50 µM z-VAD-fmk for the times indicated. **E** Procaspase-8 was immunoprecipitated from pooled fractions, and co-immunoprecipitation of FADD, RIPK1, and TRAIL-R2 was studied by western blotting.

We next sought to characterize the activation platform for caspase-8 under these conditions. In untreated and treated HCT-116 (Bax/Bak)^−/−^ cells, caspase-8 was primarily located in the cytosol (Fig. 4A, B). We then performed gel filtration of cytosolic extracts to study if caspase-8 integrates into high molecular weight complexes, as has been described for non-canonical cytosolic activation platforms such as ripoptosomes [6, 7]. These experiments need to be conducted in presence of pan-caspase inhibitor zVAD-fmk to prevent processing and loss of caspase-8 from such complexes as well as the downstream activation of effector caspases and their possible feedback cleavage on caspase-8 [6, 7]. Gel filtration of cytosolic fractions indeed demonstrated that bortezomib treatment induced the formation of high molecular weight complexes containing caspase-8 between 36 h and 40 h of bortezomib treatment (Fig. 4C, D; corresponding to ~2 MDa size). High molecular weight fractions containing caspase-8 also contained FADD as a key adapter protein required for canonical caspase-8 activation, cFLIP as well as RIPK1 (Fig. 4C, D). It is notable that both cFLIP and RIPK1 were found in high molecular weight fractions of untreated cells to then disappear transiently upon treatment before reappearing together with caspase-8 in these fractions (Fig. 4C, D). Immunoprecipitating caspase-8 from pooled eluted fractions co-precipitated FADD, RIPK1, and surprisingly also TRAIL-R2, a death receptor involved in extrinsic apoptosis and that has previously also been implicated in the activation of caspase-8 upon ER stress [8] (Fig. 4E). Consistent with previous reports [21, 22], the overall amounts of cFLIP and TRAIL-R2 accumulated upon proteasome inhibition; the amounts of FADD and RIPK1 instead decreased and could partially be stabilized by caspase inhibition (Fig. S3A, B). Overall, these results, therefore, identify that proteasome inhibition induces the formation of large caspase-8 activation platforms that accumulate in the cytosol and that contain an otherwise largely membrane-associated death receptor.

### TRAIL-R2 is essential for caspase-8 activation upon proteasome inhibition

**Fig. 5:**
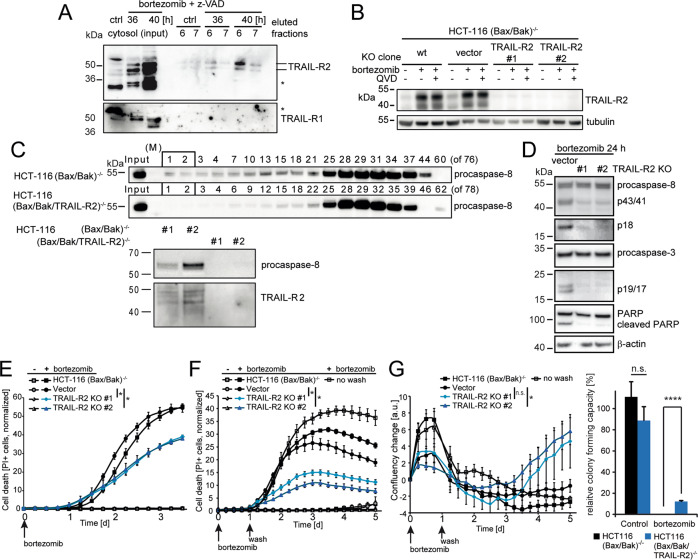
TRAIL-R2 is essential for caspase-8 activation upon proteasome inhibition. **A** Cytosolic high molecular weight fractions of untreated or treated HCT-116 (Bax/Bak)^−/−^ cells were analyzed for the presence of TRAIL-R1 and TRAIL-R2 by western blotting. *Marks unspecific bands. **B** HCT-116 (Bax/Bak)^−/−^ cells, as well as clones obtained by CRISPR/Cas9 gene targeting, were studied for TRAIL-R2 expression. Cells were treated with 100 nM bortezomib ±20 µM Q-VD-OPh. Tubulin served as a loading control. **C** HCT-116 (Bax/Bak)^−/−^ and HCT-116 (Bax/Bak/TRAIL-R2)^−/−^ cells were treated with 100 nM bortezomib for 48 h. Proteins from soluble cytosolic fractions were separated by molecular weight and the presence of procaspase-8 was analyzed (upper panels). High molecular weight fractions were compared for the presence of procaspase-8 and TRAIL-R2 (lower panels). **D** Vector control and TRAIL-R2^-/-^ clones of HCT-116 (Bax/Bak)^−/−^ cells were treated with 100 nM bortezomib for 24 h, and processing of caspase-8, caspase-3, and PARP were analyzed by western blotting. **E** Cell death kinetics measured by PI uptake using time-lapse imaging. Cells were treated with 100 nM bortezomib or left untreated. Data are means ± sd of *n* = 3 independent experiments, each conducted in triplicates with three quantified fields of view per well (*p < 0.05, *t*-tests with Bonferroni corrections). **F** Cell death kinetics measured as in (**E**), with bortezomib, wash out after 24 h. (*p < 0.05, *t*-tests with Bonferroni corrections). **G** Confluency changes in arbitrary units for cell populations treated as in (F) (*p < 0.05, *t*-tests with Bonferroni corrections). The bar graph shows the associated relative colony formation capacity of untreated and treated cells. Data are means + sd from *n* = 3 independent experiments (****p < 0.0001, *t*-test).

TRAIL-R2 was previously implicated in non-canonical caspase-8 activation upon glucose starvation and ER stress [8, 10, 23]. We therefore next studied if TRAIL receptors are crucial for caspase-8 activation and cell death induction upon proteasome inhibition. In line with the co-immunoprecipitation data (Fig. 4E), we detected TRAIL-R2 in high molecular weight fractions (Fig. 5A), corresponding to those in which caspase-8 and FADD can be found (Fig. 4D). In contrast, TRAIL-R1 could not be identified (Fig. 5A). TRAIL-R2 and its accumulation could be detected upon proteasome inhibition, whereas these signals were absent in cells in which we eliminated TRAIL-R2 expression by CRISPR/Cas9 targeting of *TNFRSF10B* (Fig. 5B). Cells lacking TRAIL-R2 failed to recruit caspase-8 into high molecular weight complexes (Fig. 5C), failed to activate caspases-8 and -3, and failed to cleave PARP (Fig. 5D). Loss of TRAIL-R2 also substantially reduced proteasome inhibitor-induced cell death (Fig. 5E). Since bortezomib is rapidly cleared in vivo (580 nM peak serum concentration; 13 nM after 4 h; 2–3 nM after 72 h; approx. concentrations calculated from [24]), we also examined cell death and proliferation after drug washout at 24 h. TRAIL-R2 deficiency substantially reduced overall cell death (Fig. 5F) and promoted cell re-growth (Fig. 5G). Associated assays for colony formation capacity likewise demonstrated that loss of TRAIL-R2 allowed individual cells to escape cell death and resume proliferation (Fig. 5G). Additional depletion of TRAIL-R1 did not reduce cell death further (Fig. S4A, B). Taken together, these data provide evidence that the presence of TRAIL-R2 is a critical requirement for caspase-8 activation and apoptotic cell death upon proteasome inhibition in otherwise highly apoptosis-resistant cells.

## Discussion

Here, we report that proteasome inhibition triggers TRAIL-R2-dependent apoptosis in cells incapable of activating the mitochondrial apoptosis pathway. In response to proteasome inhibition, TRAIL-R2 ultimately accumulates in cytosolic complexes that contain FADD and RIPK1, and which serve to activate caspase-8. The cytosolic complexes reach molecular weights in the megadalton range, but obviously, also smaller aggregates could already provide caspase activity. Loss of TRAIL-R2 entirely abrogates caspase-dependent cell death following proteasome inhibition, highlighting the crucial role of this receptor in triggering programmed cell death when canonical intrinsic apoptosis is suppressed.

The relevance of caspase-8 activation outside of classical ligand-induced extrinsic apoptosis has been demonstrated in various scenarios in which distinct caspase-8 activation platforms form [6–9, 11]. RIPK1 is a key component of caspase-8 activating complexes (ripoptosomes) forming upon cIAP depletion, etoposide-induced genotoxic stress, and thapsigargin-induced ER stress, however, with its kinase activity apparently obsolete in the latter scenario [6, 7, 9]. In our initial characterizations, we observed that cisplatin-induced genotoxicity induces considerable amounts of cell death in Bax/Bak deficient cells, associated with moderate caspase-8 and -3 processing (Fig. 1C), which could be attributable to these activation complexes. RIPK1-dependent caspase-8 activating complexes also form as cells enter mitosis, and here limited RIPK1 and caspase-8 activities contribute to genome stability [25]. While RIPK1 can also counteract caspase-8 activation in TNF receptor signaling [26], RIPK1 does not seem to prevent premature apoptosis induction under conditions of proteasome inhibition or to prolong the time during which cells can try to resolve the stress scenario, since RIPK1 depletion failed to enhance cell death in response to bortezomib.

During canonical extrinsic apoptosis, at least six TRAIL receptors need to oligomerize to form DISCs [27], which is followed by caspase-8 activation resulting from dimerization within filaments that form through DED-domain interactions [28–30]. How TRAIL-R2 oligomerizes intracellularly upon proteasome inhibition remains unresolved so far. Since ligand binding is not required, the accumulation of TRAIL-R2, possibly within cytosolic aggresomes or, preceding this, within ER or Golgi membranes [31], may suffice to induce oligomerization and caspase-8 activation. In line with this, forced overexpression of TRAIL receptors suffices to induce apoptosis [32]. In addition, it was recently shown that TRAIL-R2 pre-assembles into trimers without the need for TRAIL binding [33], so that higher-order oligomers may easily form. It was furthermore recently suggested that misfolded proteins can bind to and activate TRAIL-R2 [31], a mechanism that might contribute to TRAIL-R2 activation also in the scenario of proteasome inhibition. While TRAIL death receptors typically are membrane proteins, also non-membranous protein pools have been described and found to have crucial roles in cellular signaling besides canonical extrinsic apoptosis [34–37]. Overall, however, more detailed studies on TRAIL receptor trafficking in the above-described scenarios would help to better understand the spatiotemporal regulation and function of TRAIL receptor signaling.

Canonical extrinsic apoptosis can result in rapid caspase-8 activation and cell death within a few hours, whereas caspase-8 activation in response to proteasome inhibition requires significantly longer to manifest. Upon proteasome inhibition, cells likely first mount pro-survival stress responses before succumbing to unresolved stress. One likely player at conditions of proteasome inhibition might be cFLIP variants, all of which are short-lived proteins [38]. In previous work, we found that cFLIP accumulates as a consequence of proteasome inhibition and thereby delays extrinsically induced caspase-8 activation [21]. Another notable difference to extrinsic apoptosis is that in contrast to the fast and complete processing of the entire cellular caspase-8 pool, upon proteasome inhibition only modest amounts of the total caspase-8 pool are recruited to the intracellular activation platforms (the majority of procaspase-8 remains a cytosolic monomer). It is currently unknown what limits the further recruitment of procaspase-8 to the activation platform, but it would undoubtedly be helpful to understand if and which processes or conformations are involved in restricting more efficient caspase-8 activation and processing. Similar limitations in caspase-8 recruitment into activation platforms were also reported during ripoptosome-induced apoptotic cell death [6, 7].

Besides its role in cell death induction, additional roles for caspase-8 have recently been identified. For example, the presence of caspase-8 but not its activity is required in FADD- and RIPK1-containing complexes that form in cells stimulated with TRAIL and that induce the production of inflammatory cytokines [39]. If caspase-8 exerts a similar scaffold function within ligand-independent activation platforms remains unknown so far. In other complexes, caspase-8 plays a non-apoptotic role in DNA damage sensing and genomic stability [25, 40]. Additionally, caspase-8 activity can contribute to cell cycle progression by overcoming the G_2_/M checkpoint [41]. These pro-survival roles of caspase-8 highlight that cells need to be able to fine-tune caspase-8 activation and probably also their downstream susceptibility to caspase-8 activities [42]. In contrast to self-amplifying apoptosis execution signaling, dose-response relations dominate during TRAIL-induced caspase-8 activation, even when signaling is fully geared towards apoptosis induction [14, 43]. Furthermore, caspase-8 activity can contribute to preventing unwanted cell death by cleaving RIPK1 and thereby disassembling caspase-8 activation platforms [44]. Overall, it, therefore, cannot be excluded that limited TRAIL-R2-dependent caspase-8 activation upon proteasome inhibition might initially promote pro-survival signaling and only is translated into a death-inducing signal over time since active caspase-8 can no longer be degraded.

Since proteasome inhibitors such as bortezomib and carfilzomib are in clinical use [13], knowledge on their potential to induce cell death in cancer cells that are highly resistant to Bax/Bak-dependent mitochondrial apoptosis might offer translational perspectives. Molecular characterizations and indices that allow assessing resistance to mitochondrial apoptosis prior to treatment onset [45, 46] might assist in identifying patients that would benefit from proteasome inhibitor-based treatments that could circumvent thresholds set for example by high expression of anti-apoptotic Bcl-2 family members, such as Bcl-2, Bcl-xL, and Mcl-1 [47, 48]. Similarly, the cellular capacity to activate caspase-8 in response to proteasome inhibition could provide a scenario in which targeted therapeutics that neutralize anti-apoptotic Bcl-2 family members could offer means to enhance cancer cell susceptibility to canonical intrinsic apoptosis [49]. Likewise, drug development strategies geared at antagonizing cFLIP could provide means by which caspase-8 activation upon proteasome inhibition could be enhanced and more reliably be directed towards apoptosis execution [48].

## Supplementary information

Supplemental Figs Legends

Supplemental Fig 1

Supplemental Fig 2

Supplemental Fig 3

Supplemental Fig 4
