# Supplementary material for: Proteasome inhibition triggers the formation of TRAIL receptor 2 platforms for caspase-8 activation that accumulate in the cytosol
Source: Cell Death Differ. 2021 Aug 5;29(1):147–55. doi: 10.1038/s41418-021-00843-7 (PMC8738721; doi:10.1038/s41418-021-00843-7)
Supplement: Supplementary file 1 — Supplemental Figs Legends [file 41418_2021_843_MOESM1_ESM.docx]

**Supplemental Information 1:**

**(A)** Proteasomal LLVYase activity was measured in HCT-116 cells treated with 100 nM bortezomib.

**(B)** Parental MEFs (par) and MEF (Bax/Bak)-/- cells were treated with intrinsic apoptosis triggers (100 μg/ml 5-fluorouracil (5-FU), 40 μM cisplatin, 3 μM tunicamycin (tunica), 100 nM bortezomib) as indicated. Cell death was measured by PI uptake. Data are means ± s.d. from triplicate samples of a representative experiment. Immunoblot insert confirms loss of Bax and Bak in HCT-116 (Bax/Bak)-/- cells. β-actin served as loading control. *p<0.05 (multiple t-test).

**(C)** Cells were treated for 48 h as in (B), and cleavage of procaspase-8 and procaspase-3 was analyzed by western blotting. β-actin served as loading control.

**(D)** Quantification of FRET-negative MEF (Bax/Bak)-/- cells expressing an IETD FRET probe after treatment with 100 nM bortezomib ± 20 μM z-VAD-fmk. Data show mean + s.d. (* p<0.05, Sidak’s multiple comparisons test).

**(E)** Specific cell death in HCT-116 (Bax/Bak)-/- cells treated with 100 nM bortezomib and in which RIPK1 expression was depleted. RIPK1 depletion was confirmed by western blotting. Data are means ± s.d. from n=3 independent experiments (ns, not significant, unpaired t-tests).

**(F)** Cell death in HCT-116 (Bax/Bak)-/- cells treated with 100 nM bortezomib and in which PGAM5 was depleted. PGAM5 depletion was confirmed by western blotting. Data are means ± s.e.m. from n=4 independent experiments (* p<0.05, unpaired t-tests).

**Supplemental Information 2:**

**(A)** HCT-116 (Bax/Bak)-/- cells were left untreated or treated with 100 nM bortezomib, with or without addition of each 1 μg/ml TRAIL-R1:Fc, TRAIL-R2:Fc, and TNF-R1:Fc neutralizing antibodies (ABs) for 48 hours. Procaspase-8 processing was analyzed by western blotting. β-actin served as loading control.

**(B)** HCT-116 (Bax/Bak)-/- cells were treated with 100 ng/ml TRAIL ± TRAIL-R1:Fc, TRAILR2: Fc, and TNF-R1:Fc neutralizing antibodies (each 1 μg/ml) for 24 h. Neutralizing antibodies abrogated caspase processing. β-actin served as loading control.

**(C)** HCT-116 (Bax/Bak)-/- cells were treated with 100 nM bortezomib with or without addition of 10 mM 3-methyladenine (3MA) for 48 h. Cleavage of procaspase-8 was analyzed by western blotting. Porin served as loading control.

**(D)** HCT-116 (Bax/Bak)-/- cells were transfected with scrambled (scr) or ATG5-directed siRNA (siAtg5). Cells were treated with 100 nM bortezomib and procaspase-8 processing was analyzed by western blotting. β-actin and porin served as loading controls.

**Supplemental Information 3:**

**(A)** Temporal profiles of cFLIP species following treatment with 100 nM bortezomib. 50 µM zVAD was added as indicated. Actin served as loading control. *unknown or unspecific bands.

**(B)** Temporal profiles of RIPK1, FADD and TRAIL-R2 following treatment with 100 nM bortezomib. 50 µM zVAD was added as indicated. GAPDH served as loading control.

**Supplemental Information 4:**

**(A)** HCT-116 (Bax/Bak)-/- cells (wt) and TRAIL-R2-deficient clones #1 and #2 were transfected with scramble siRNA or TRAIL-R1-directed siRNA. After 24 h, cells were treated with 100 nM bortezomib ± 5 μM Q-VD-OPh for 48 h. Data are means ± s.d. from n=3 independent experiments.

**(B)** Western blotting confirms depletion of TRAIL-R1 expression after siRNA transfection. Tubulin serves as loading control.
