# Supplementary figures and images for: Proteasome inhibition triggers the formation of TRAIL receptor 2 platforms for caspase-8 activation that accumulate in the cytosol
Source: Cell Death Differ. 2021 Aug 5;29(1):147–55. doi: 10.1038/s41418-021-00843-7 (PMC8738721; doi:10.1038/s41418-021-00843-7)

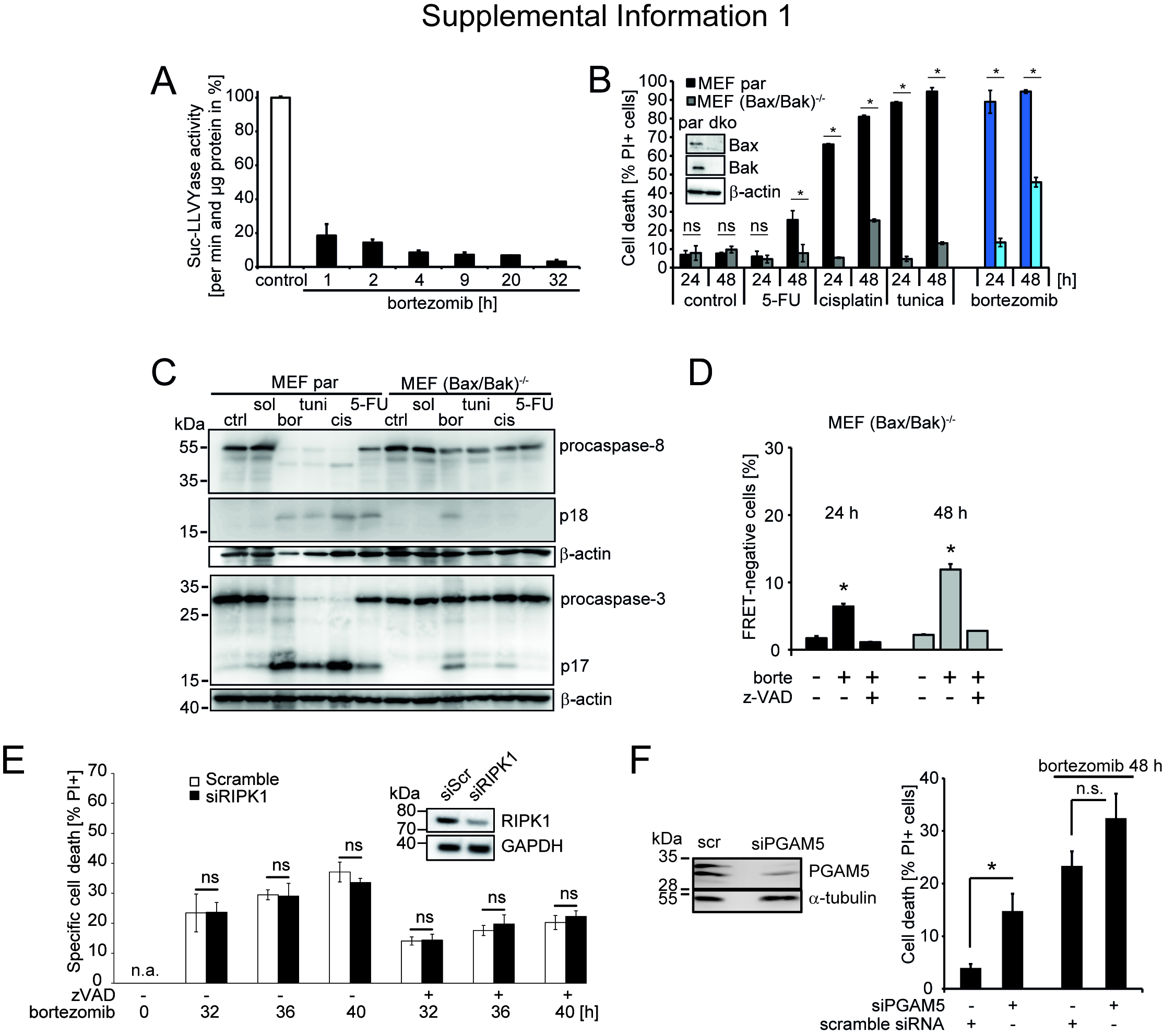

Supplement: Supplementary file 2 — Supplemental Fig 1 [file 41418_2021_843_MOESM2_ESM.tif]

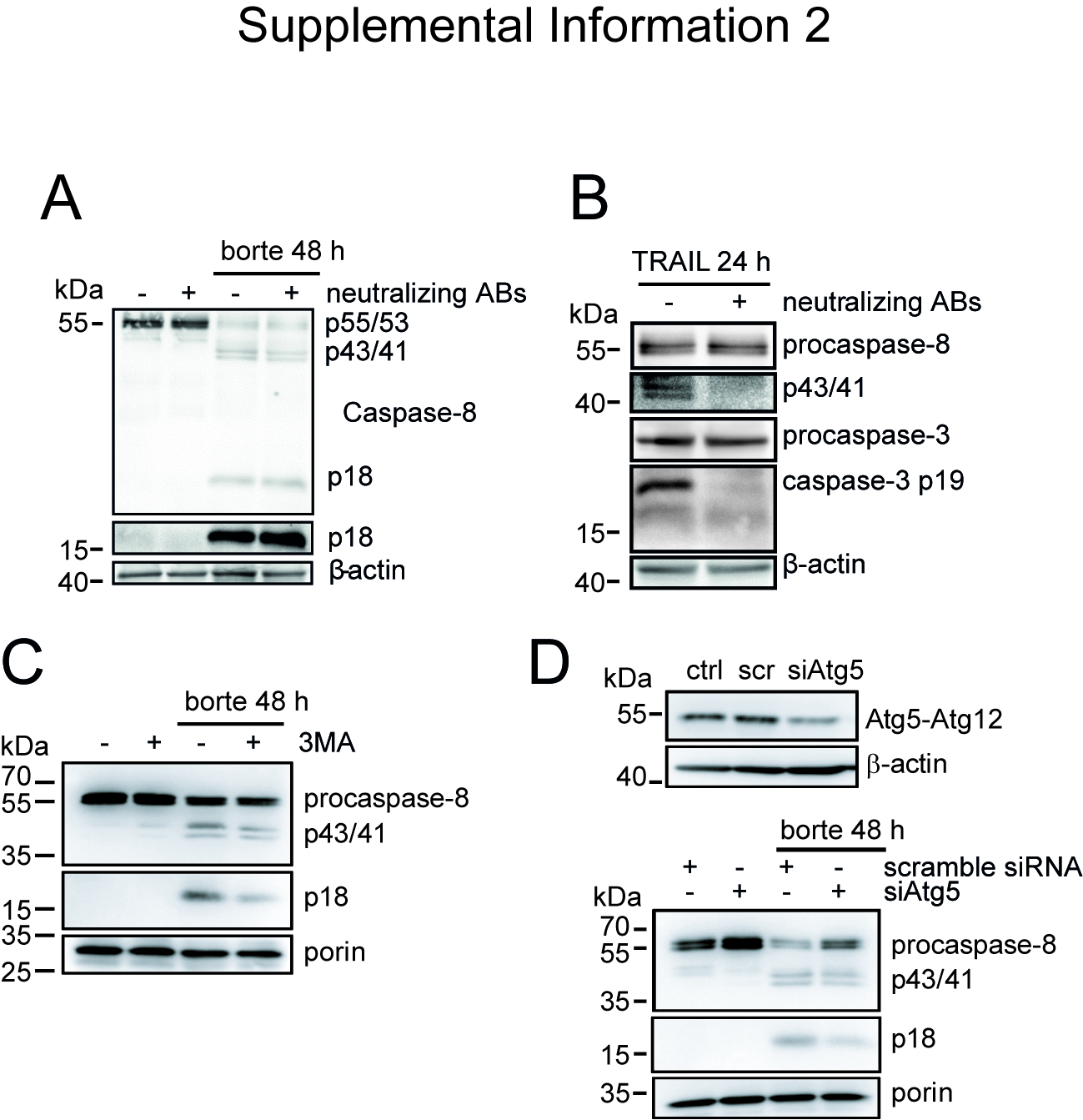

Supplement: Supplementary file 3 — Supplemental Fig 2 [file 41418_2021_843_MOESM3_ESM.tif]

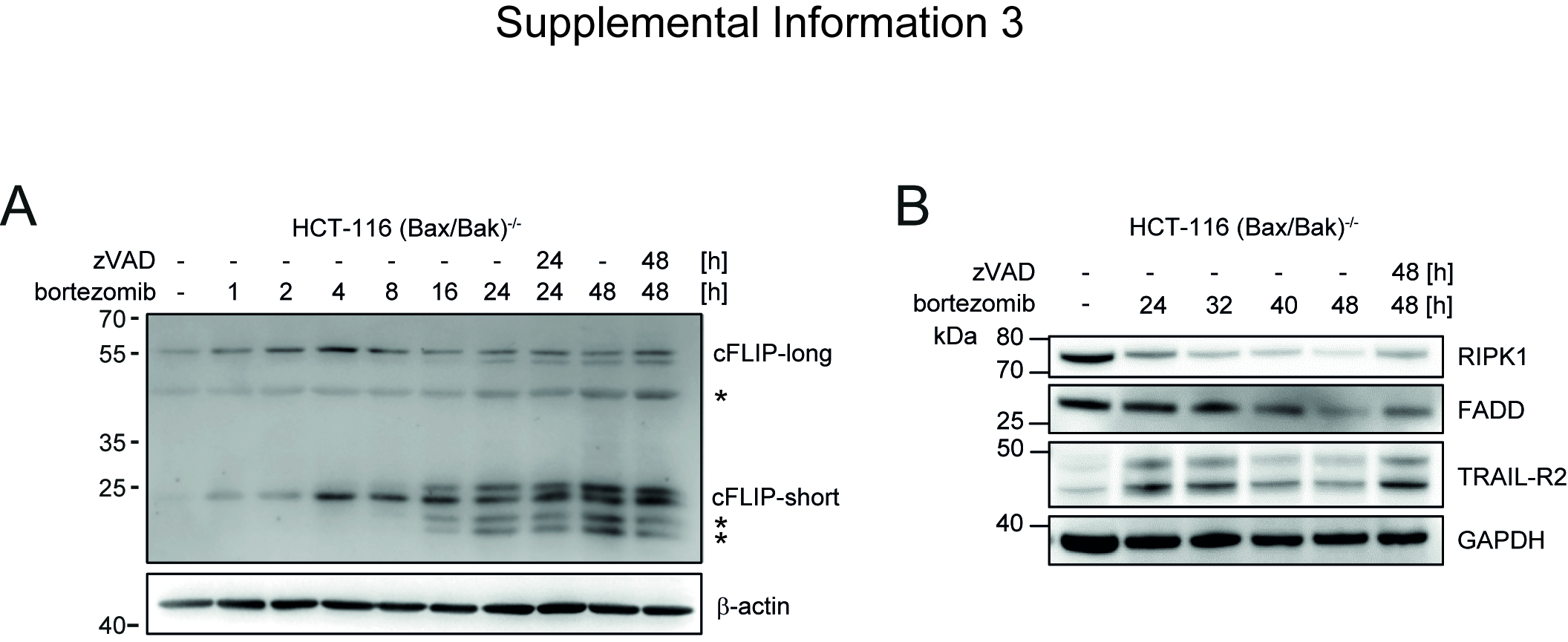

Supplement: Supplementary file 4 — Supplemental Fig 3 [file 41418_2021_843_MOESM4_ESM.tif]

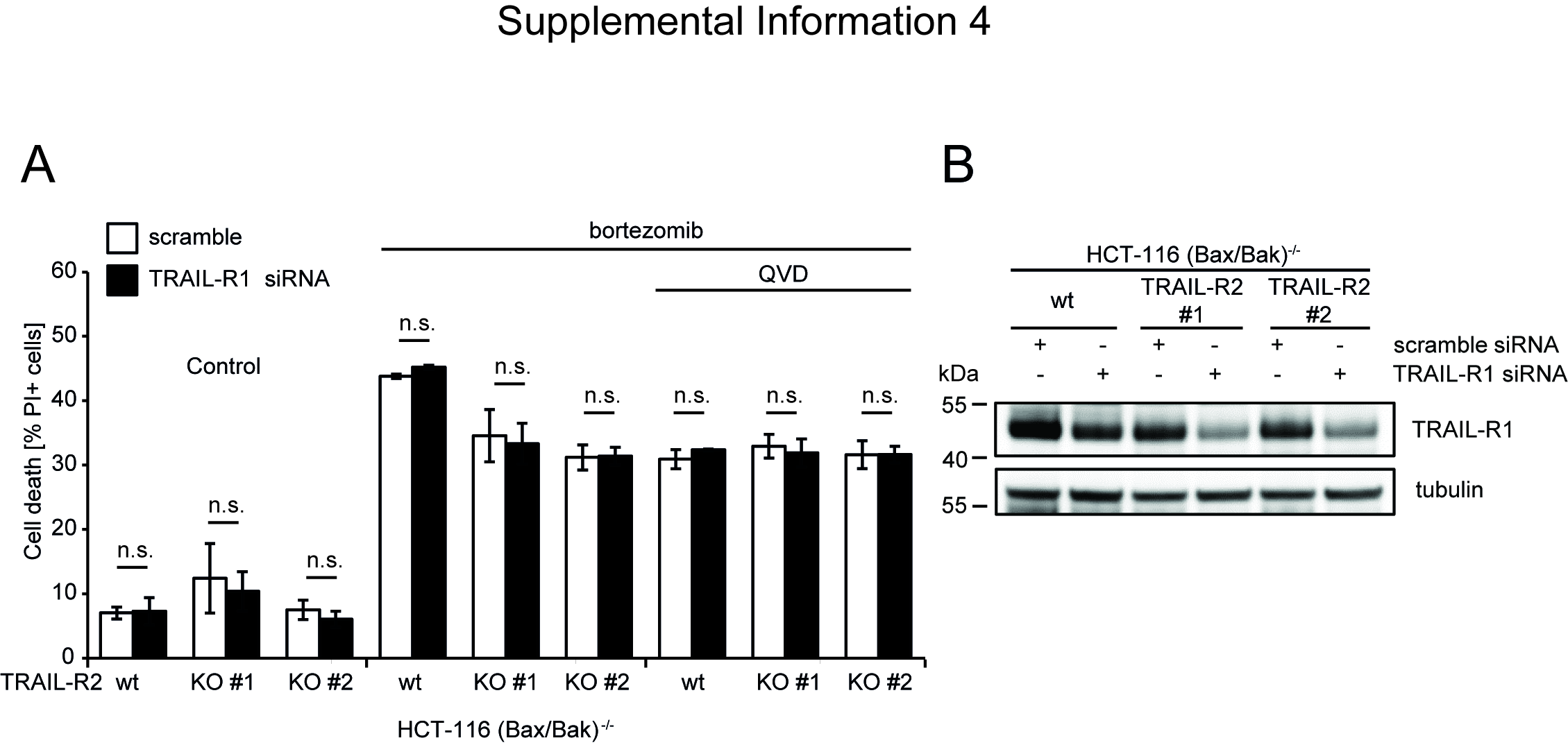

Supplement: Supplementary file 5 — Supplemental Fig 4 [file 41418_2021_843_MOESM5_ESM.tif]
